# High-Elevation Populations of Montane Grasshoppers Exhibit Greater Developmental Plasticity in Response to Seasonal Cues

**DOI:** 10.3389/fphys.2021.738992

**Published:** 2021-11-04

**Authors:** Julia M. Smith, Rory S. Telemeco, Bryan A. Briones Ortiz, César R. Nufio, Lauren B. Buckley

**Affiliations:** ^1^Department of Biology, University of Washington, Seattle, WA, United States; ^2^Department of Biology, California State University, Fresno, Fresno, CA, United States; ^3^School of Aquatic and Fishery Sciences, University of Washington, Seattle, WA, United States; ^4^Howard Hughes Medical Institute, Chevy Chase, VA, United States; ^5^University of Colorado Museum of Natural History, University of Colorado, Boulder, Boulder, CO, United States

**Keywords:** climate change, development, physiology, temperature-size rule, thermal sensitivity

## Abstract

Populations of insects can differ in how sensitive their development, growth, and performance are to environmental conditions such as temperature and daylength. The environmental sensitivity of development can alter phenology (seasonal timing) and ecology. Warming accelerates development of most populations. However, high-elevation and season-limited populations can exhibit developmental plasticity to either advance or prolong development depending on conditions. We examine how diurnal temperature variation and daylength interact to shape growth, development, and performance of several populations of the montane grasshopper, *Melanoplus boulderensis*, along an elevation gradient. We then compare these experimental results to observed patterns of development in the field. Although populations exhibited similar thermal sensitivities of development under long-day conditions, development of high-elevation populations was more sensitive to temperature under short-day conditions. This developmental plasticity resulted in rapid development of high elevation populations in short-day conditions with high temperature variability, consistent with their observed capacity for rapid development in the field when conditions are permissive early in the season. Notably, accelerated development generally did not decrease body size or alter body shape. Developmental conditions did not strongly influence thermal tolerance but altered the temperature dependence of performance in difficult-to-predict ways. In sum, the high-elevation and season-limited populations exhibited developmental plasticity that enables advancing or prolonging development consistent with field phenology. Our results suggest these patterns are driven by the thermal sensitivity of development increasing when days are short early in the season compared to when days are long later in the season. Developmental plasticity will shape phenological responses to climate change with potential implications for community and ecosystem structure.

## Introduction

Recent climate warming has advanced phenology including reproduction and adult emergence in a large (∼80%, Parmesan, 2006) proportion of species, but the fitness consequences of these phenological shifts are often unclear (Forrest and Miller-Rushing, 2010). Laboratory rearing experiments reveal developmental plasticity in growth and development rates in response to environmental cues (Atkinson, 1996; West-Eberhard, 2003). Developmental plasticity varies as a function of life-history strategy and the environment to which populations are adapted (Forrest, 2016). High temperatures generally reduce development time and decrease adult size in insects and other ectotherms due to development being more thermally sensitive than growth (Bale et al., 2002; Sgro et al., 2016). However, short growing seasons at high latitudes or high elevations can drive a reversal of this temperature-size rule, such that higher rearing temperatures produce larger adult size (Dingle et al., 1990; Hodkinson, 2005; Berner and Blanckenhorn, 2006). What are the implications of these growth and developmental tradeoffs for phenological shifts observed in the field? The combination of greater warming and greater thermal sensitivity of development for high elevation populations may flatten phenological gradients occurring along elevational clines (Chmura et al., 2018; Vitasse et al., 2018; Nufio and Buckley, 2019).

We examine how growth and development rates of montane grasshopper populations respond to seasonal cues- diurnal temperature variation and daylength- as well as how these changes affect adult performance. We then interpret our experimental, laboratory observations in light of historical, and recent phenology observed for the grasshopper populations in the field. We focus on grasshopper populations spanning a 1,500 m elevation gradient along the 40th N parallel in Boulder County, CO, United States. Weekly survey data from the Gordon Alexander Project reveal that the first appearance of adults has generally advanced between initial surveys in 1959–1960 and resurveys conducted since 2006 (Nufio et al., 2010). Phenological advancements and phenological variation across elevation were most pronounced for early-season species, such as our focal species (Nufio and Buckley, 2019). Progression through juvenile developmental stages has also generally advanced, but development proceeds more slowly and developmental stages persist longer in warm conditions for the high-elevation, season-limited populations of early-season species (Buckley et al., 2015; Nufio and Buckley, 2019). Broader phenology can increase overlap among species with potential implications for interactions in communities (Buckley et al., 2021).

Under controlled laboratory conditions, we further test the hypothesis that higher elevation populations exhibit greater developmental plasticity due to occupying more variable, season-limited environments. Daylength often cues developmental rate when conditions are permissive in time constrained environments (Bradshaw and Holzapfel, 2007). We build on a previous study for a generalist, late-season grasshopper species, *M. sanguinipes* (Buckley et al., 2015). Higher-elevation *M. sanguinipes* populations displayed greater phenotypic plasticity in development rate in response to developmental temperature than lower-elevation populations (Buckley et al., 2015). This resulted in higher-elevation populations advancing their phenology more than lower-elevation populations in warm conditions (Buckley et al., 2015). Studies on univoltine, high-latitude damselflies found that short daylengths consistent with seasonal time limitations accelerated development (Sniegula et al., 2016; Norling, 2018). However, they found less, rather than our hypothesized more, developmental plasticity among high-latitude populations (Sniegula et al., 2016).

Here we examine how the development plasticity of an early-season species, *Melanoplus boulderensis*, responds to naturalistic variation in both temperature and daylength. We intended these factors as naturalistic seasonal cues but note that greater temperature variability also exposes grasshoppers to higher temperatures and fixed daylengths throughout development would not be experienced in nature. *M. boulderensis* has a restricted, montane distribution (Otte, 2012) and is cool adapted (Buckley et al., 2013b; Buckley and Nufio, 2014). Dispersal ability is limited by short wings and the species exhibits genetic differentiation along the elevation gradient (Slatyer et al., 2020). The species is univoltine, which excludes phenological advancements from increasing fitness by adding an additional seasonal generation.

We assess growth and development responses of three elevationally distinct populations to temperature variability and daylength. In addition to the hypothesis of greater developmental plasticity for high-elevation populations, we hypothesized that long-day conditions experienced at later developmental stages, indicative of a release from seasonal time constraints, would slow development. To further assess the fitness consequences of growth and development, we assess plasticity in thermal sensitivity and the temperature dependence of performance resulting from rearing conditions. These data allow us to test for potential trade-offs between accelerated development and performance, as well as for potential matching of performance to developmental conditions. For example, we might expect grasshoppers that rapidly develop to incur tradeoffs that reduce their peak performance capacity as adults, or for grasshoppers that develop under more variable temperatures to display more generalist thermal performance. Finally, we review field phenology across the elevation gradient in light of our findings on how temperature and daylength influence development rates.

## Materials and Methods

### Rearing Experiments

We examined development rates among *M. boulderensis* populations inhabiting three montane or subalpine sites along the 40th N parallel in Boulder County, CO: A1 (2,195 m, 40.01N, 105.37W), B1 (2,591 m, 40.02N, 105.43W), C1 (3,048 m, 40.03N, 105.55W) (descriptions^1^). The sites are all grassy meadows, with somewhat denser vegetation at the lower-elevation sites. *M. boulderensis* overwinters in an egg diapause with eggs deposited in pods just below the soil surface. The species is a generalist consumer of forbs so vegetation phenology is not expected to substantially constrain its phenology.

Eggs were collected by allowing individual, labeled females, collected from the three field sites in mid-summer, to oviposit in damp sand, and then sieving the sand. Eggs were stored in damp vermiculite within 2oz polyurethane containers. The surface was periodically coated with 0.25% methyl-p-hydroxy benzoate to inhibit fungal or microbial growth. The eggs first developed for 3 weeks in incubators at near ambient conditions (25–30°C), which is required to enable an obligate diapause (Dingle et al., 1990), and were then stored in diapause conditions at 2°C for ∼110 days. Following diapause, the eggs were moved to our experimental treatments within incubators and individuals were maintained in these treatments throughout hatching and development to adulthood (details below). Upon hatching, the egg containers were enclosed within rectangular 2.25L polyurethane containers and lettuce and wheat bran were provided. The grasshoppers were reared together until they reached 3rd instar. Subsequently, grasshoppers were reared individually in 0.47L polyurethane containers, which were changed every other day and supplied with romaine lettuce and wheat bran. We checked for eclosion when containers were changed. For newly eclosed grasshoppers, we noted the date and stage, and measured mass (g, Mettler Toledo AL104 balance). For adults, we additionally measured pronotum and femur length (mm) using digital calipers.

*Melanoplus boulderensis* post-diapause eggs and juvenile instars from each of the three populations were reared in factorial combinations of high (24 ± 4°C, HV treatment) or low (24 ± 2°C, LV treatment) temperature variability and long (14 h:10 h light:dark cycle) or short (12 h:12 h light:dark cycle) daylength. The daylengths were chosen to correspond to mid-summer versus early (March) or late (September) conditions. Temperature varied diurnally as a step function aligned with the 12 h:12 h photoperiod. Temperatures were chosen based on previous fixed temperature experiments, to allow for rapid development while maintaining high survival. A side-effect of our thermal treatment design was that faster development occurred at higher temperatures in the high-variance thermal treatment than the low-variance treatment even though their mean temperatures were the same. This difference is most easily interpreted by calculating the constant temperature equivalent (CTE) which is the median developmental temperature after accounting for development speed increasing with temperature (for CTE equations and additional details see Georges, 1989; Georges et al., 2004; Telemeco et al., 2013). The CTE for our treatments differed by 1°C, with the HV treatment having a CTE of 26°C and the LV treatment having a CTE of 25°C. Grasshoppers were reared in Percival I-36VL incubators with 32W fluorescent bulbs (Phillips F32T8/TL741). There was no indication that the grasshoppers were able to use the lights to thermoregulate. The final analysis included grasshoppers that survived to maturity from populations at 2,195 m (*n* = 104), 2,591 m (*n* = 63), and 3,048 m (*n* = 69). We assessed adult age and mass for an average of 10 individuals for each treatment, population, and sex combination (median = 9, range 3–19).

### Thermal Sensitivity of Reared Grasshoppers

Most individuals were measured for all thermal traits with measurements occurring in the following order: hopping performance at four temperatures, feeding performance at three temperatures, preferred body temperatures (PBT), critical thermal minimum (CT_min_), and critical thermal maximum (CT_max_). We selected this order to minimize the potential for earlier measurements to bias later measurements, although this potential cannot be completely removed.

#### Hopping Performance

To assess the temperature dependence of hopping performance, we acclimated grasshoppers for 1 h at one of four temperatures (10, 17, 25, or 35°C) in incubators (same type as for rearing treatments) after which we immediately measured the distance of five jumps. To control for potential exposure order effects, we measured each grasshopper at each temperature in one of four orders (10-35-25-17; 25-17-10-35; 17-10-35-25; or 10-17-35-25). All measurements occurred across 2 days. After acclimation, grasshoppers were removed individually from the incubators and immediately placed in the center of the experimental arena at room temperature. The arena consisted of a 1.8 m × 1.8 m sheet of fabric with a checkered pattern at an interval of 2.5 cm (methods follow Harrison et al., 1991). Hopping was induced by manual prodding if necessary. We marked the position of the grasshopper after each of five jumps and subsequently recorded the *x* and *y* locations to an *x* and *y* resolution of 2.5 cm. We assessed hopping performance for an average of 7 individuals for each treatment, population, and sex combination (median = 7, range 3–18).

#### Feeding Performance

Grasshoppers were fasted prior to each feeding trial for 12 h, a sufficient period to complete digestion and absorption (Harrison and Fewell, 1995), and provided with a damp paper towel for humidity during trials. Feeding trials were conducted at three temperatures (10, 20, and 40°C). We assessed feeding performance for an average of 8 individuals for each treatment, population, and sex combination (median = 7.5, range 3–17).

The order of temperature trials was randomized. Grasshoppers were acclimated to the test temperature for 1 h prior to being provided with organic, baby romaine leaves at the start of two consecutive feeding periods each day. The first feeding period lasted for 2 h (reflecting rates of ingestion and of crop and mid-gut filling) and the later feeding period lasted an additional 6 h [reflecting rates of ingestion, crop filling and gut throughput; (Harrison and Fewell, 1995)]. The initial feeding trials commenced between 07:00 and 09:00 h. We used a flatbed scanner (Canon LiDE 100) to photograph the leaves before and after each of the feeding trials. We estimated leaf areas using ImageJ software^2^.

#### Preferred Body Temperatures and Critical Thermal Limits

We first measured PBT using a thermal gradient constructed on an aluminum sheet (0.125″ × 24″ × 48″). We placed one end in an ice bath and the other on a hotplate (Springate and Thomas, 2005), which created a temperature gradient spanning 5–50°C. Grasshoppers were placed within 5-cm-wide lanes created by corrugated plastic dividers running longitudinally across the thermal gradient. A clear acrylic lid was then placed above the gradient with holes for circulation and thermocouple measurements, and the grasshoppers were allowed to acclimate for 30 min. We then used an Extech type K thermocouple to monitor the thermal gradient and record the temperatures associated with the position of grasshoppers every 10 min over a 50-min period (Forsman, 2000; Springate and Thomas, 2005). During the acclimation period, grasshoppers moved freely throughout their lane on the thermal gradient before reducing activity. Most grasshoppers spent the duration of the observation period resting in one position. We assessed PBT for an average of 7 individuals for each treatment, population, and sex combination (median = 7, range 2–15).

We measured critical thermal limits, CT_min_ and CT_max_, which were defined as the lower and upper temperatures at which the grasshoppers were no longer able to right themselves. Grasshoppers were placed individually into 50ml centrifuge tubes, which were slowly (∼0.2°C min^–1^) cooled or heated in a water bath. Given that warming rates may influence estimates of critical thermal limits, we chose an intermediate rate of warming (Chown et al., 2009). To minimize stress, we first cooled body temperatures for CT_min_ estimates and then began heating body temperatures for CT_max_ estimates at least an hour after the conclusion of the CT_min_ assays. We assessed CT_min_ for an average of 5 individuals for each treatment, population, and sex combination (median = 3.5, range 0–11). For CT_max_ the average was 4 (median = 3.5, range 0–10).

### Field Phenology

For comparison to our experimental results, we analyzed historic (1958–1960) and recent (2006–2016) phenology data for the same *M. boulderensis* populations that sourced individuals for our rearing experiment as part of the Gordon Alexander Project. Surveys consisted of 1 person-hour of sweep netting and 0.5 person-hours of searching for adults and juveniles that may have been missed by sweep netting (Nufio et al., 2010; Nufio and Buckley, 2019). Data and analyses are as in Nufio and Buckley (2019).

We calculated degree days as the accumulated product of time and temperature above the lower developmental temperature (LDT). The calculation employed a single-sine approximation (Allen, 1976) based on daily minimum and maximum temperatures and a fixed spacing of 12 h between temperature minima and maxima. We used daily maximum and minimum temperature data from weather stations at our study sites (McGuire et al., 2012; Nufio and Buckley, 2019). For the field analysis, we calculated degree days based on air temperature to avoid assumptions regarding thermoregulatory behavior, radiation, windspeed, and soil temperatures. We use an LDT of 0°C, which corresponds to an estimate based on rearing *M. boulderensis* in constant temperatures and regressing development time against temperature (Trudgill, 1995). This differs from previous analyses (Nufio et al., 2010; Nufio and Buckley, 2019) that used an LDT of 12.0°C based on a fit to field phenology data pooled across multiple species. Our estimation of degree days for field populations are intended as an approximate translation of environmental temperature into physiological time.

We used a development index (DI), which represents the average development stage of the population and ranges from 1 (all first instars) to 6 (all adults), to describe the developmental stage of communities sampled through field surveys. We also used the DI to estimate the timing of adulthood, because DI generally exhibits a smooth increase through the season whereas counts of individual development stages can be variable. DI also allows interpolating between the weekly survey intervals. We quantified phenology both in terms of day of year (doy) and growing degree days (GDDs). We fit a spline (R function smooth.spline) to DI data for each combination of species, site, and year. We used the splines to estimate the timing of adulthood as the doy or GDDs when DI = 5.5 (R predict function). We selected DI = 5.5 as it tended to approximately correspond to the inflection point before the DI curve reached the asymptote at DI = 6.

### Analyses

Our analyses primarily used linear mixed-effects (LME) models and ANOVAs in R (lmer function from Lme4 library; Bates et al., 2007). We used an LME model to evaluate the (categorical) effects of temperature variance, photoperiod, site, sex, and their interactions up to the fourth order. We checked for normality of the response variables and subsequently assumed a normal distribution. We used Akaike information criterion corrected for small sample size (AICc) to compare the full model to models restricted to a subset of terms and interactions. We used model selection whereby models were preferred if ΔAICc > 2. However, as described below, for some dependent variables we selected slightly less-preferred models for ease of comparison to other dependent variables. Models included the identity of the grasshopper’s mother as a random effect except where indicated.

The full model was preferred for development time (days from removal from diapause conditions to adulthood). Several simpler models were preferred to the full model for adult mass, but we used the full model to facilitate comparison to the model for development time. We repeated the analyses for development time and mass, additionally including developmental stage (instar) as a numeric predictor (3rd to 6th = adult) to assess whether effects on age and mass shifted over development in a repeated-measures ANOVA. We assessed correlations between femur length and mass^1/3^ and between pronotum length and mass^1/3^ to determine whether rearing conditions altered body shape.

We used the model for PBT receiving the most support, which included only the main effects. The model we selected for critical thermal minimum and maximum included temperature variation, photoperiod, and the interaction between the two. No single model received support for CT_min_ or CT_max_, so we chose the most complex model with ΔAICc < 2. We also omitted the random effect since it led to slightly higher AICc values and singularity issues.

To examine rearing effects on hopping and feeding performance, the full model was preferred based on AICc scores. For hopping performance, we included a random effect to account for the grasshopper measured (nested within the identity of the mother) since each individual performed five replicate hops. We accounted for test temperature as a second-order polynomial for hopping, due to the unimodal shape and improved AICc scores, and as a linear term for feeding given that we had only three test temperatures and no obvious non-linearity of response. Our dependent variable was consumed leaf area scaled to the mass of the grasshopper over the full 8 h test period (visual inspection showed similar trends existed at 2 h).

We quantified the temperature dependence of growth and development rates using Q10 values, which describe the shift in rates with a 10-degree temperature increase as *Q10* = (*R*_2_/*R*_1_)^[10/(*T*_2_-*T*_1_)], where, *R*_1_ and *R*_2_ are rates at temperatures *T*_1_ and *T*_2_ (Seebacher et al., 2015). *Q10* values represent the effects of temperature on physiological rates that are normalized for comparison, and are commonly used to compare the thermal sensitivity of processes such as growth and development. We normalized *R*_1_ = 1 at *T*_1_ = 24°C, the mean rearing temperature. We estimated the number of development days, *d*, to reach adulthood as *d* = *c*/[*Q10*^(*T*_*var*_/10)+*Q10*^(−*T*_*var*_/10)], where, *T*_*var*_ is the temperature variance from *T*_1_ = 20°C and *c* is a constant that accounts for 12 h at each temperature and assumes that a fixed number of units of development are required to reach adulthood. Similarly, we estimate the Q10 for growth using the following expression for adult mass, *M* (g) as *M* = *c d* [*Q10*^(*T*_*var*_/10)+*Q10*^(−*T*_*var*_/10)], where, *d* is the number of days of development. We estimate development and growth Q10s for each site, daylength, and sex using the generalized least squares model fitting function gnls() from the nlme package for R (Pinheiro et al., 2020) and plot the estimates as relative Q10s by normalizing the highest Q10 estimate to 1. We then used these Q10 values to graphically assess the effects of elevation-of-origin and daylength on the thermal sensitivity of development rate.

For the field phenology data, we used linear mixed-effects (LME) models and ANOVAs in R to examine how the development index responds to the 2nd-degree polynomial of day of year or cumulative degree days, season warmth, site, and their interaction. We controlled for survey year as a random variable.

## Results

### Growth and Development

**FIGURE 1:**
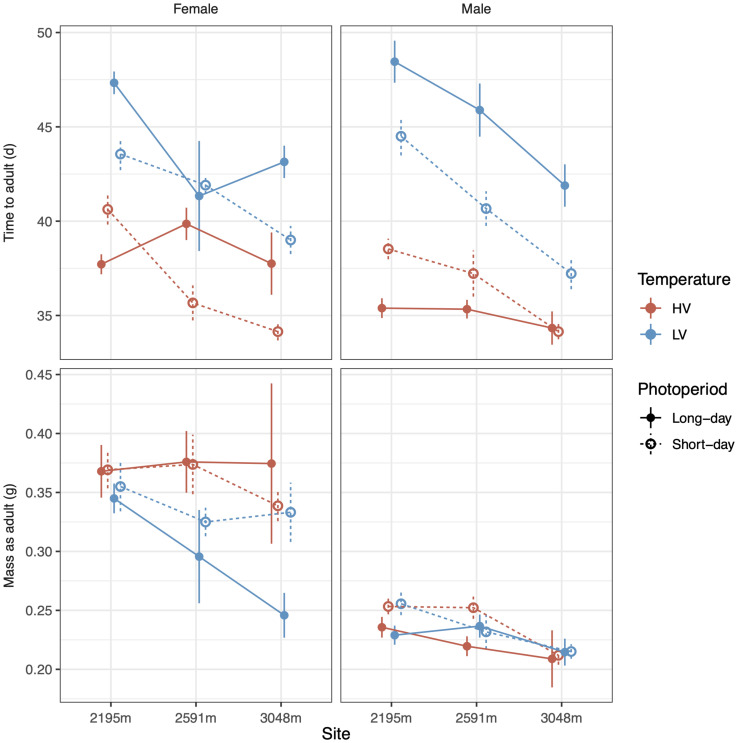
Time to adulthood **(top row)** and adult mass **(bottom row)** vary as a function of photoperiod, temperature variance, site, and sex. High temperature variance (red color) leads to faster development, and males develop faster than females (column) across sites (*X*-axis). Photoperiod (line type) interacts with temperature to determine development rates. Low temperature variance at higher elevation sites leads to lower mass, especially in females. Error bars represent standard error.

Temperature variation and photoperiod interacted with site and sex to determine time to adulthood (Figure 1, top row). The high-temperature variation treatment (HV) which had a CTE 1°C higher than the low-temperature variation treatment (LV) substantially accelerated development (Table 1, top row, coefficients: Supplementary Table 1, model plot: Supplementary Figure 1). Long-days accelerated development in HV conditions but decelerated development in LV conditions, particularly at low elevations and for males (model plot: Supplementary Figure 1). Although higher elevation populations had reduced development times for most treatments, this effect of elevation-of-origin disappeared when animals were reared under HV, long-day conditions (Figure 1). Significant lesser-order interactions are largely consistent with the four-way interaction (Table 1; coefficients: Supplementary Table 1). Analyzing time to each instar indicates that the effects of photoperiod and temperature variability on development become most apparent late in development (Supplementary Figure 2). The four-way interaction described above also significantly interacts with instar (Supplementary Tables 2, 3).

**TABLE 1:** Wald 3 ANOVA for linear mixed effects models of grasshopper time to adulthood (days) and mass at adulthood (g).

	Time to adult			Mass at adult		
	χ^2^	*df*	*p*	χ^2^	*df*	*p*
(Intercept)	2348.7	1	<0.001***	768.6	1	<0.001***
Sex	6.4	1	0.011*	56.3	1	<0.001***
Site	1.6	2	0.460	0.1	2	0.933
Temperature	66.8	1	<0.001***	1.2	1	0.273
Photoperiod	6.4	1	0.011*	0.0	1	0.943
Sex:Site	1.4	2	0.505	1.0	2	0.619
Sex:Temperature	4.7	1	0.031*	0.3	1	0.568
Site:Temperature	17.8	2	<0.001***	8.4	2	0.015*
Sex:Photoperiod	0.0	1	0.850	0.4	1	0.506
Site:Temperature	16.3	2	<0.001***	1.3	2	0.533
Temperature:Photoperiod	15.1	1	<0.001***	0.1	1	0.774
Sex:Site:Temperature	8.8	2	0.012*	6.0	2	0.049*
Sex:Site:Photoperiod	6.0	2	0.0497*	0.3	2	0.857
Sex:Temperature:Photoperiod	0.0	1	0.928	0.0	1	0.991
Site:Temperature:Photoperiod	17.0	2	<0.001***	5.9	2	0.053
Sex:Site:Temperature:Photoperiod	12.4	2	0.002**	3.6	2	0.164

“Temperature” refers to temperature variance and “Site” indicates three source locations at different elevations. Stars indicate significant effects (*: *p* < 0.05, **: *p* < 0.01, and ***: *p* < 0.001).

**FIGURE 2:**
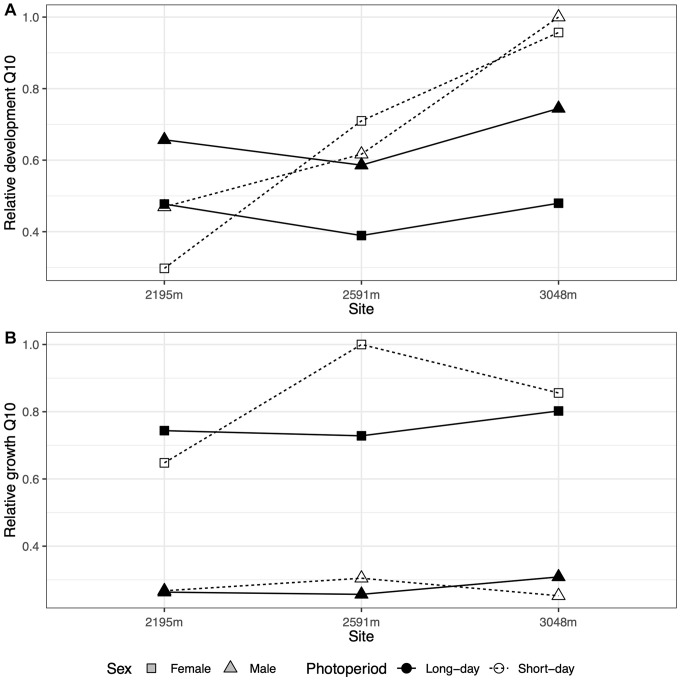
**(A)** The relative thermal sensitivity (Q10) of development (normalized from 0 to 1) is relatively flat across site elevations for long-day conditions (filled symbols and solid lines) but increases with elevation for short-day conditions (open symbols and dashed lines) for both sexes (symbols). **(B)** The relative thermal sensitivity (Q10) of growth does not vary consistently with photoperiod.

Estimating the thermal sensitivity of development using Q10 values suggests similar thermal sensitivity across elevation for long-day conditions (Figure 2A). However, Q10 values, and thus physiological responsiveness to temperature increases, are estimated to increase with elevation under short-day conditions, which suggest the capacity for rapid growth when conditions are permissive. Consistent with the Q10 estimates, high-elevation populations in short-day conditions exhibited the fastest development (Figure 1). By contrast, Q10s estimated for growth were relatively flat across elevation regardless of daylength, although females had higher Q10s than males (Figure 2B).

Shifts in developmental rates and times influenced adult mass, but in a manner that diverges from the simple expectation that prolonged development increases mass. Interestingly, there is not a significant linear relationship between development time and adult mass across all individuals from all treatments (coefficient = 0.0014 ± 0.0011 SE, *t*_[1,234]_ = 1.3, *p* = 0.2, *r*^2^ = 0.003). The LV thermal treatment, which prolonged development (Figure 1, top row), did not increase mass, and, in fact, substantially decreased mass in females from higher-elevation sites (Figure 1, bottom row, χ^2^_2_ = 6.0, *p* < 0.05, Table 1 and Supplementary Figure 3). Comparing just the lowest and highest elevation sites suggests that lower masses at higher elevations induced by the LV thermal treatment were extenuated by long-day conditions (*t* = 2.4, *p* < 0.05, Supplementary Table 1 and Supplementary Figure 4). Body shape was not influenced by temperature variability or daylength, as adult femur length and pronotum length each have a strong linear relationship with mass^1/3^ (femur coefficient = 11.7 ± 0.8 SE, *t* = 15.0, *p* < 0.001, *r*^2^ = 0.49; pronotum coefficient = 7.0 ± 0.4 SE, *t* = 18.4, *p* < 0.001, *r*^2^ = 0.59). Analyzing the mass of each instar does not show significant interactions between instar and these variables, except that the mass differential between males and females emerges and grows as development proceeds (see Supplementary Figure 5 and Supplementary Tables 2, 3).

### Thermal Sensitivity

**FIGURE 3:**
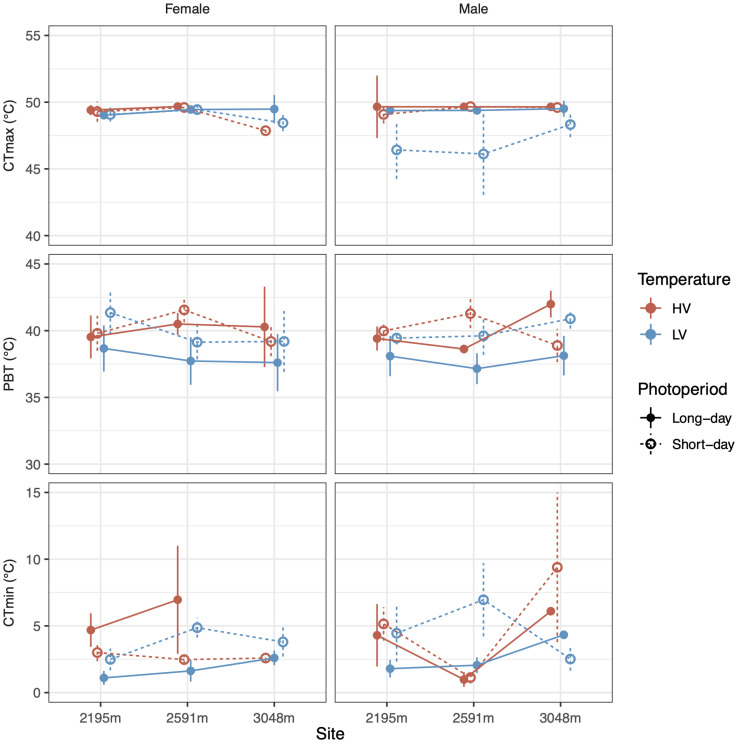
Photoperiod (line type) and temperature variance (colors) alter CT_min_ **(bottom row)** but not CT_max_ **(top row)** of male and female (columns) grasshoppers from different populations (*X*-axis). Mean preferred body temperatures **(middle row)** of lab-reared grasshoppers do not differ significantly based on sex, photoperiod, temperature variance, or population. Error bars represent standard error.

Preferred body temperature was not significantly affected by our rearing treatments, but there were non-significant trends for both the LV (χ^2^_1_ = 3.0, *p* = 0.08) and long-day treatments (χ^2^_1_ = 3.1, *p* = 0.08) reducing preferred temperature by up to ∼3°C (Figure 3 and Table 2; coefficients: and Supplementary Table 4). LV, long-day rearing decreased grasshopper CT_min_ by up to ∼4°C (mean = 4.46°C, F = 4.1, *p* < 0.05), while we found no effects of rearing treatments on the grasshoppers’ CT_max_ (mean = 49.2°C, Figure 3 and Table 2; coefficients: Supplementary Table 5).

**TABLE 2:** Wald 3 ANOVA of our linear mixed effects model of (A) preferred body temperature, and (B) CTmin and CTmax (°C) as a function of temperature variance, photoperiod, sex, site, and interactions.

(A)								
	χ^**2**^				***df***			***p***

(Intercept)	4143.1				1			<0.001***
Sex	0.0				1			0.854
Site	0.1				2			0.966
Temperature	3.0				1			0.081
Photoperiod	3.1				1			0.077

**(B)**								
	**CT_min_**				**CT_max_**			
	***Sum Sq***	***df***	***F value***	***p***	***Sum Sq***	***df***	***F value***	***p***

(Intercept)	468.9	1	44.9	<0.001***	46629.0	1	12215.9	<0.001***
Temperature	89.1	1	8.5	0.004**	0.4	1	0.1	0.737
Photoperiod	4.2	1	0.4	0.529	1.4	1	0.4	0.544
Temperature:Photoperiod	43.0	1	4.1	0.045*	4.2	1	1.1	0.295
Residuals	1107.2	106			385.5	101		

Stars indicate significant effects (*: *p* < 0.05, **: *p* < 0.01, and ***: *p* < 0.001).

**FIGURE 4:**
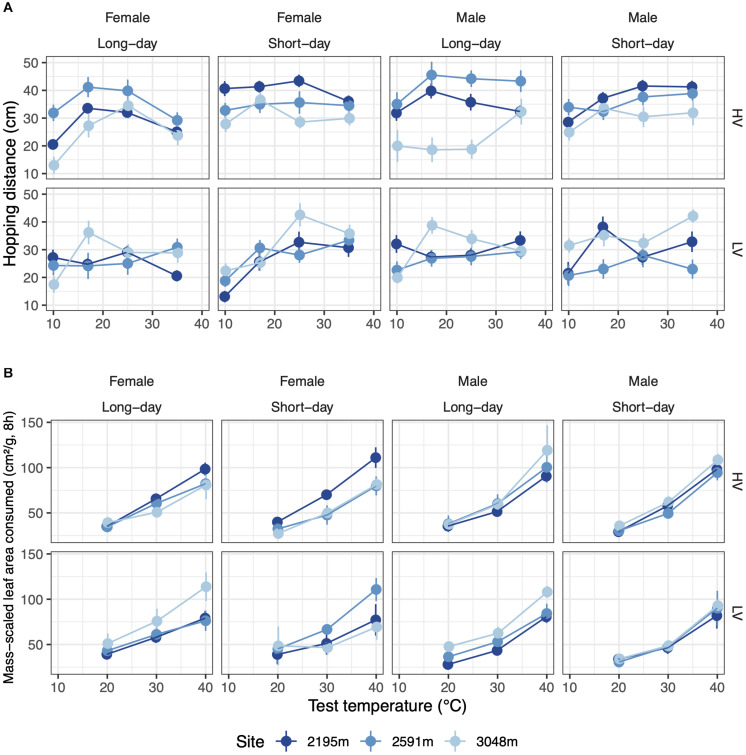
**(A)** The temperature dependence of hopping performance varies with developmental temperature variation (rows) and photoperiod (columns). **(B)** The temperature dependence of feeding performance varies with temperature variability (rows) but not photoperiod (columns). Error bars represent standard error.

The temperature sensitivity of hopping performance responded to both temperature variability and photoperiod during rearing in a manner that depended on sex and site: the female thermal performance curve was narrow (indicated by TestTemp^2^ in Supplementary Table 6) when reared under HV, long-day conditions, and was especially narrow for the highest elevation site (five-way interaction: χ^2^_4_ = 13.8, *p* < 0.05, Figure 4A and Table 3). Hopping performance was typically highest when reared under short days (χ^2^_1_ = 11.7, *p* < 0.001), especially for grasshoppers reared in the HV thermal treatment (χ^2^_1_ = 14.9, *p* < 0.001, Table 3). Low-temperature hopping performance declined when grasshoppers were reared in both HV, long-day conditions and LV, short-day conditions (test temperature x temperature variance x photoperiod: χ^2^_2_ = 22.1, *p* < 0.001, Figure 4A and Table 3; coefficients: Supplementary Table 6).

**TABLE 3:** Wald 3 ANOVA of our linear mixed effects model of hopping distance (cm) and feeding performance (cm^2^/g).

	Hopping distance			Leaf area consumed		
	χ^2^	*df*	*p*	χ^2^	*df*	*p*
(Intercept)	0.3	1	0.603	8.6	1	0.003**
f(TestTemp)	26.4	2	<0.001***	114.5	1	<0.001***
Sex	6.1	1	0.013*	0.1	1	0.704
Site	4.2	2	0.120	1.4	2	0.478
Temperature	4.0	1	0.046*	2.5	1	0.113
Photoperiod	11.7	1	<0.001***	0.1	1	0.778
f(TestTemp):Sex	3.8	2	0.148	1.0	1	0.328
f(TestTemp):Site	5.9	4	0.205	3.6	2	0.162
Sex:Site	3.8	2	0.146	2.4	2	0.304
f(TestTemp):Temperature	6.6	2	0.037*	5.0	1	0.026*
Sex:Temperature	0.0	1	0.927	1.9	1	0.166
Site:Temperature	0.0	2	0.979	1.2	2	0.537
f(TestTemp):Photoperiod	6.5	2	0.039*	0.7	1	0.395
Sex:Photoperiod	9.6	1	0.002*(*)	0.5	1	0.495
Site:Photoperiod	2.0	2	0.373	0.4	2	0.801
Temperature:Photoperiod	14.9	1	<0.001***	0.0	1	0.884
f(TestTemp):Sex:Site	10.6	4	0.0314*	6.4	2	0.042
f(TestTemp):Sex:Temperature	2.3	2	0.317	2.2	1	0.137
f(TestTemp):Site:Temperature	7.3	4	0.122	5.1	2	0.077
Sex:Site:Temperature	4.3	2	0.115	2.8	2	0.253
f(TestTemp):Sex:Photoperiod	16.2	2	<0.001***	0.2	1	0.621
f(TestTemp):Site:Photoperiod	6.1	4	0.194	0.5	2	0.785
Sex:Site:Photoperiod	4.2	2	0.123	1.3	2	0.511
f(TestTemp):Temperature: Photoperiod	22.1	2	<0.001***	0.3	1	0.558
Sex:Temperature:Photoperiod	2.8	1	0.092	0.5	1	0.493
Site:Temperature:Photoperiod	1.4	2	0.494	3.4	2	0.181
f(TestTemp):Sex:Site:Temperature	13.8	4	0.008**	5.6	2	0.060
f(TestTemp):Sex:Site:Photoperiod	13.7	4	0.008**	1.3	2	0.527
f(TestTemp):Sex:Temperature: Photoperiod	13.8	2	0.001**	0.2	1	0.679
f(TestTemp):Site:Temperature: Photoperiod	8.1	4	0.088	8.5	2	0.015*
Sex:Site:Temperature:Photoperiod	4.1	2	0.132	3.4	2	0.183
f(TestTemp):Sex:Site:Temperature: Photoperiod	13.8	4	0.008**	4.5	2	0.105

f(TestTemp) = TestTemp + TestTemp^2^ in the case of hopping distance and f(TestTemp) = TestTemp in the case of feeding performance. Stars indicate significant effects (*: *p* < 0.05, **: *p* < 0.01, and ***: *p* < 0.001).

Grasshopper feeding rate increased up to our highest test temperature (40°C) for all rearing conditions and populations (Figure 4B). This positive effect of temperature on feeding rate was strongest for grasshoppers raised in HV conditions (χ^2^_1_ = 5.0, *p* < 0.05, Figure 4B and Table 3). Additionally, as elevation increased, the temperature dependence of males’ feeding increased while that of females decreased, leading to a performance differential at high test temperatures (χ^2^_2_ = 6.4, *p* < 0.05, Table 3). With increasing elevation, LV grasshoppers’ feeding performance at high test temperatures transitioned from worse than HV grasshoppers to better (χ^2^_2_ = 8.5, *p* < 0.05, Table 3; coefficients: Supplementary Table 6).

### Field Phenology

**FIGURE 5:**
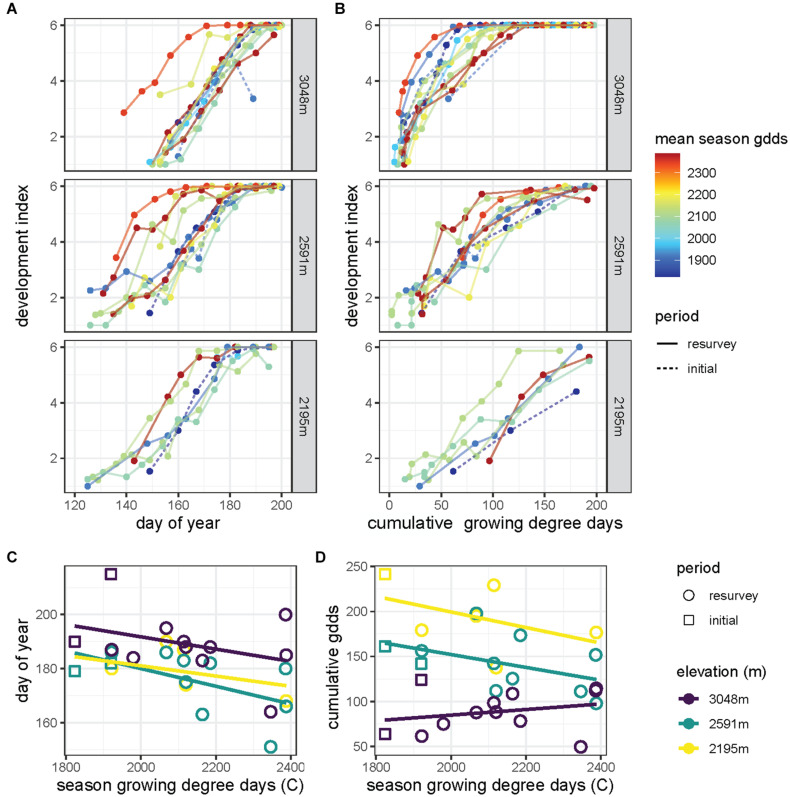
The development index, which represents the average developmental stage of the population and ranges from 1 (all first instars) to 6 (all adults), increases **(A)** with day of year and **(B)** as growing degree-days accumulate through the season for sites across elevation (rows). Lines correspond to years of the initial survey (dashed) and resurvey (solid) with colors indicating the seasonal growing degree-days averaged along the elevational gradient (red, warm years to blue, cool years). Adult phenology of *Melanoplus boulderensis* (estimated using the development index) quantified as **(C)** day of year and **(D)** cumulative growing degree days (gdds) varies as a function of seasonal cumulative growing degree days. We distinguish years during the initial survey (squares) and resurvey (circles) for each site elevation (color).

At each elevation, grasshoppers developed and matured into adults faster during warmer seasons, although development through late instars appeared to slow for some warm seasons (Figure 5A). Season warmth interacted with day of year to determine the developmental progression (χ^2^_2_ = 22.3, *p* < 0.001), but the response to season warmth did not vary significantly across sites. Several warm seasons exhibited slow development and several cool seasons exhibited rapid development in response to the accumulation of growing degree days at the 3,048 m site (Figure 5B). Phenological differences with season warmth were less apparent at the lower elevation sites. The accumulation of degree days, season warmth, and site significantly interacted in determining the progression of development (χ^2^_4_ = 18.0, *p* < 0.001).

The day of year of adulthood accelerated in warmer seasons (F_[2,23]_ = 6.0, *p* < 0.001) and varied across sites (*F*_[__2,23__]_ = 5.0, *P* < 0.05), but the response to season warmth was similar across sites (*F*_[__2,23__]_ = 0.1, *P* = 0.87, Figure 5C). The cumulative degree days at adulthood did not shift significantly in warmer seasons (*F*_[__2,23__]_ = 2.7, *P* = 0.11, Figure 5D), with the relationship being particularly flat for the 3,048 m site. The cumulative degree days at adulthood varied across sites (*F*_[__2,23__]_ = 28.1, *p* < 0.001) but did not significantly interact with season warmth (*F*_[__2,23__]_ = 1.7, *P* = 0.21, Figure 5D). See Nufio and Buckley (2019) for further analysis and comparison with other sites and species.

## Discussion

Our rearing experiment builds on past inferences, based on field phenology, of greater developmental plasticity in response to temperature for season-limited high-elevation populations of *M. boulderensis* grasshoppers compared to low-elevation populations. Rearing temperature and daylength interacted to cue plastic variation in developmental rate consistent with phenological patterns observed in the field. Somewhat surprisingly, we did not detect evidence for trade-offs between accelerated development and other fitness-relevant traits such as body size, thermal tolerance, and performance, although thermal sensitivities for performance were affected by rearing treatment in complex ways.

Grasshoppers from high-elevation populations generally developed into adults more rapidly than those from low-elevation populations, but the size of this effect depended strongly on rearing treatment. For example, the treatment that most closely modeled mid-summer, high-elevation conditions (long-day, HV) was least affected by population of origin, with all populations developing quickly. By contrast, we observed the greatest effect of elevation-of-origin for the treatment most closely modeling springtime, low-elevation conditions (short-day, LV), with high-elevation animals developing much more rapidly than low-elevation animals. These differences appear to be driven by short daylengths, typical of spring, increasing the thermal sensitivity of development (i.e., Q10) for the highest-elevation population but not the lower-elevation populations. This plasticity suggests that high-elevation populations have the capacity to facultatively increase their rate of development when conditions are permissive early in the season.

Additionally, grasshoppers reared in the HV temperature treatment developed more rapidly than those reared in the LV treatment. This effect can be partially explained by HV animals developing at warmer temperatures with a constant temperature equivalent (CTE) 1°C higher than animals reared in the LV treatment. However, a 1°C average difference in developmental temperature appears insufficient to fully explain the ∼10-day difference in development time between HV and LV treatments. For example, in *M. sanguinipes*, another grasshopper species from this community, a 6°C difference in constant rearing temperature (24°C vs. 30°C) is needed to induce a similar effect on development time (Buckley et al., 2015). These results suggest that differences in temperature variation between the HV and LV treatments affected development rate independent of the direct effect of temperature on development, although additional data are needed to confirm this conclusion. Observations of low *M. boulderensis* survival in high, constant temperatures in preliminary rearing experiments led to our examination of fluctuating temperatures. We intended thermal variance as an indicator of seasonality but selected a constant mean temperature for tractability. Shifting both the mean and variance of treatments so that the CTE is controlled across treatments could help disentangle these effects. Further, in most studies of developmental plasticity including ours there is a need to consider more realistic environmental variation both to capture the variation during a single day and to represent environmental shifts during the course of development (Sniegula et al., 2016; Burggren, 2018).

Examining daylength in this study allowed us to refine previous conclusions that developmental plasticity at high elevation extended development when environmental conditions allowed (Buckley et al., 2015; Nufio and Buckley, 2019). The thermal sensitivity of development (Q10s) suggests a role of photoperiod in accelerating development when days are short, indicative of early- or late- season conditions. While the consequences are similar, this suggests selection to complete development in season-limited environments rather than selection to extend development when conditions allow and days are long. Similar roles of short photoperiod in accelerating development have been observed for other grasshopper (Dingle et al., 1990) and insect populations (Lopatina et al., 2011; Lindestad et al., 2019). However, a study including low-elevation populations of the late-season *M. sanguinipes* implicated seasonal constraints but detected daylength-x-temperature interactions in sea-level but not high-altitude populations (Dingle et al., 1990). Similarly, short days indicative of seasonal time constraints accelerated damselfly development but there was little variation in development time among high-latitude, time-constrained populations (Sniegula et al., 2016).

We did not detect a cost of accelerating development in short-day conditions such as reduced size. LV led to both delayed development and either unchanged or decreased mass, depending upon other variables, and adult mass and development time were not significantly correlated. Other studies finding similar mass invariance (Sniegula et al., 2016; Norling, 2018) suggest the need to refine understanding of tradeoffs between growth and development.

Selection for elevated thermal sensitivity in short-day conditions is often associated with ensuring the completion of a generation in the late summer in time constrained, high- elevation, or latitude environments (Dingle et al., 1990; Abrams et al., 1996; Johansson et al., 2021). However, *M. boulderensis* generally reaches adulthood in the early season before daylength declines. The latest that all members of *M. boulderensis* populations reached adulthood at any elevation across resurveys approximated the summer solstice when daylength is longest (day of year 171–173). Although cool, early-season conditions may limit development acceleration, grasshoppers can effectively use solar radiation to elevate their body temperatures once they reach sufficient size (Buckley et al., 2013a). Thus, high-elevation populations should generally develop in conditions analogous to our HV, short-day treatment which maximized development rate. *M. boulderensis* grasshoppers can lay egg pods every few days and individuals can persist as adults for at least a month at 3,048 m site (Nufio unpublished data), so faster development may allow production of more egg pods. Higher-elevation populations of *M. boulderensis* exhibit smaller eggs and clutches (Levy and Nufio, 2014; Slatyer et al., 2020), consistent with laying more clutches. Alternatively, observed developmental plasticity may enable rapid early-season development to avoid resource competition with other species when conditions are permissive. Selection for the developmental plasticity could also precede the species and its seasonal timing.

Our developmental analyses are broadly consistent with field observations of phenology (Nufio et al., 2010; Nufio and Buckley, 2019; Buckley et al., 2021). Phenological advancements of *M. boulderensis* are less apparent when considering physiological time (degree days) than calendar dates (Figure 5). This reflects the temperature dependence of development and indicates that phenology indeed responds to environmental conditions. However, comparing phenology between seasons that differ in warmth suggests greater developmental plasticity at the high-elevation site: some warm years yield slow developmental progression and some cool years yield rapid developmental progression. Daylength altering the thermal sensitivity of development can explain divergences in the relationship between growing degree days and development rate, especially at high elevation. When suitable temperatures occur early in the season when days are short, development progresses rapidly (i.e., steep slopes in Figure 5B), but when suitable temperatures only occur later in the season development progresses more slowly (i.e., shallow slopes in Figure 5B). This developmental pattern will occur regardless of the total number of growing degrees days that occur throughout the season, particularly when suitable temperatures progress late into the summer or fall. The effect of daylength on the thermal sensitivity of development can also explain why field development frequently progresses more rapidly early in the season and then slows as the population approaches all adults (i.e., asymptotic curves in Figure 5B). During the early stages of development, days are short and induce fast development, whereas days grow longer late in development thereby slowing development. Such developmental dynamics are consistent with observations of broader phenologies in warm years (Buckley et al., 2021). It is plausible that such variable developmental rates cued by daylength are adaptive, facilitating optimal development and high relative fitness, but additional experiments varying daylength across development are needed to test this hypothesis. Including more realistic seasonal photoperiod shifts is likewise needed (Sniegula et al., 2016; Norling, 2018). These studies suggest that absolute photoperiod is only part of the picture and whether the days are lengthening or shortening over time is another important cue for phenology which can help distinguish early season from late season.

As with many grasshopper species (Verberk et al., 2021), *M. boulderensis* reverses the temperature-size rule. One explanation for the reversal in grasshoppers is that warm adaptation of many physiological processes related to feeding leads to greater increases in growth than development at warm temperatures (Miller et al., 2009). This is consistent with our observations that feeding rates increase up to high temperatures. However, our Q10 analysis suggests high thermal sensitivity of development and less thermal sensitivity of growth, at least for the high elevation population. Higher developmental Q10s but roughly constant growth Q10s with increasing elevation and short-day conditions may contribute to the temperature-size rule reversal observed. Further examination of the relative slopes and intercepts of the thermal dependence of development and growth is needed to assess the mechanisms underlying the temperature-size rule for *M. bouldernesis* (Walters and Hassall, 2006).

Despite differences in the thermal sensitivity of development, we found no evidence that preferred body temperatures or critical thermal limits varied among populations. Somewhat surprisingly, CT_min_ was lower for grasshoppers reared in LV and long-day conditions which should generally model animals developing at low elevation during mid-summer when the risk of exposure to critically low temperatures is reduced. However, delayed development in these conditions may allow for broader thermal tolerance which could be useful for producing egg clutches late into fall. The greater plasticity of CT_min_ especially for the highest elevation populations is unsurprising since there is more variability in low temperatures with elevation, while hot spikes will occur regardless of elevation.

In addition to influencing development rate, temperature variance and daylength during development influenced the temperature dependence of adult hopping and feeding performance in a manner that varied among sites in difficult-to-predict ways. Some combinations of temperature variance and daylength narrowed the thermal breadth of hopping performance, but the narrowing is not readily interpretable in terms of environmental exposure. That said, development under HV, short-day conditions, which result in the fastest development across populations, also resulted in increased peak hopping performances, suggesting rearing conditions beneficial for development rate are also beneficial for performance. Increases in feeding rates with temperature up to 40°C are also consistent with faster development in HV conditions. Increased feeding performance at high temperatures in grasshoppers reared in HV conditions may be one driver of the observed developmental plasticity in development rate and hopping performance.

The evolution of rapid development at smaller size in season-limited environments is supported by fitness optimization models (Abrams et al., 1996). A study of damselflies revealed pronounced plasticity in response to photoperiod (which was changed weekly to best mimic shifting environmental conditions during growth and development), with a northern photoperiod indicative of time constraints resulting in faster development and smaller body size (Johansson et al., 2021). As with our study, the photoperiod response was more apparent for development than body mass. An analysis of genetic covariance suggested that the alignment of photoperiod with strong seasonal time constraints can promote the evolution of developmental plasticity (Johansson et al., 2021).

Our experimental results reveal complex interactions between rearing temperature, daylength, and population of origin that refute simple interpretation such as “warmer is better” or “longer is better.” Given the uncoupling of adult mass and development time, further work is needed to understand the phenotypic and fitness consequences of developmental plasticity associated with seasonal time constraints. A further complication is that thermal performance curves for hopping and feeding were affected by multiple developmental treatments and their interactions in difficult to predict ways. These observations highlight the challenges in identifying the physiological mechanisms underlying variable responses to climate change among populations and species (Buckley et al., 2018). Considering how developmental plasticity in response to seasonal cues can alter environmental responses may be central to predicting phenological and other biological implications of climate change (Forrest, 2016; Chmura et al., 2018).

## Data Availability Statement

The datasets presented in this study can be found in online repositories. The names of the repository/repositories and accession number(s) can be found below: https://github.com/HuckleyLab/GrasshopperDev.

## Author Contributions

RT and LB designed the experiment. RT led laboratory data collection. BB conducted feeding experiments. CN led field data collection. JS led analysis. JS, RT, and LB contributed to analyses and wrote the first draft of the manuscript. All authors edited the manuscript.

## Conflict of Interest

The authors declare that the research was conducted in the absence of any commercial or financial relationships that could be construed as a potential conflict of interest.

## Publisher’s Note

All claims expressed in this article are solely those of the authors and do not necessarily represent those of their affiliated organizations, or those of the publisher, the editors and the reviewers. Any product that may be evaluated in this article, or claim that may be made by its manufacturer, is not guaranteed or endorsed by the publisher.

## Footnotes

1
https://nwt.lternet.edu/explore-the-ridge

2
http://rsbweb.nih.gov/ij/
