# Supplementary material for: High-Elevation Populations of Montane Grasshoppers Exhibit Greater Developmental Plasticity in Response to Seasonal Cues
Source: Front Physiol. 2021 Nov 4;12:738992. doi: 10.3389/fphys.2021.738992 (PMC8600268; doi:10.3389/fphys.2021.738992)
Supplement: Supplementary file 1 [file Data_Sheet_1.docx]

Supplementary Material

Table S1. Estimated coefficients from our linear mixed effects model of grasshopper time to adulthood and adult mass. The intercept occurs when Sex = female, Temperature = high variance, Photoperiod = long, and Site = 2195m. Stars indicate significant effects (*: p < 0.05, **: p < 0.01, and ***: p < 0.001).


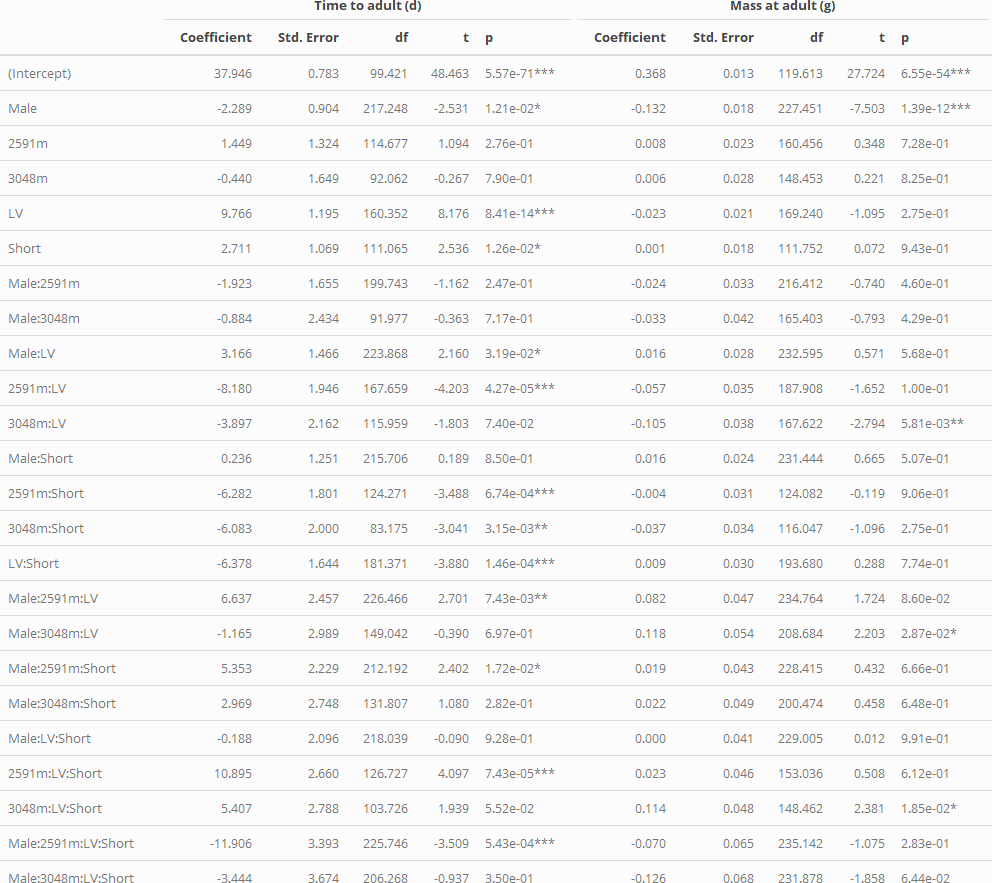


Table S2. Wald 3 ANOVA of our linear mixed effects model of grasshopper development and growth. Several higher order interactions between instar and the other variables were significant, indicating that age and mass differences emerge and grow at later instars. Stars indicate significant effects (*: p < 0.05, **: p < 0.01, and ***: p < 0.001).


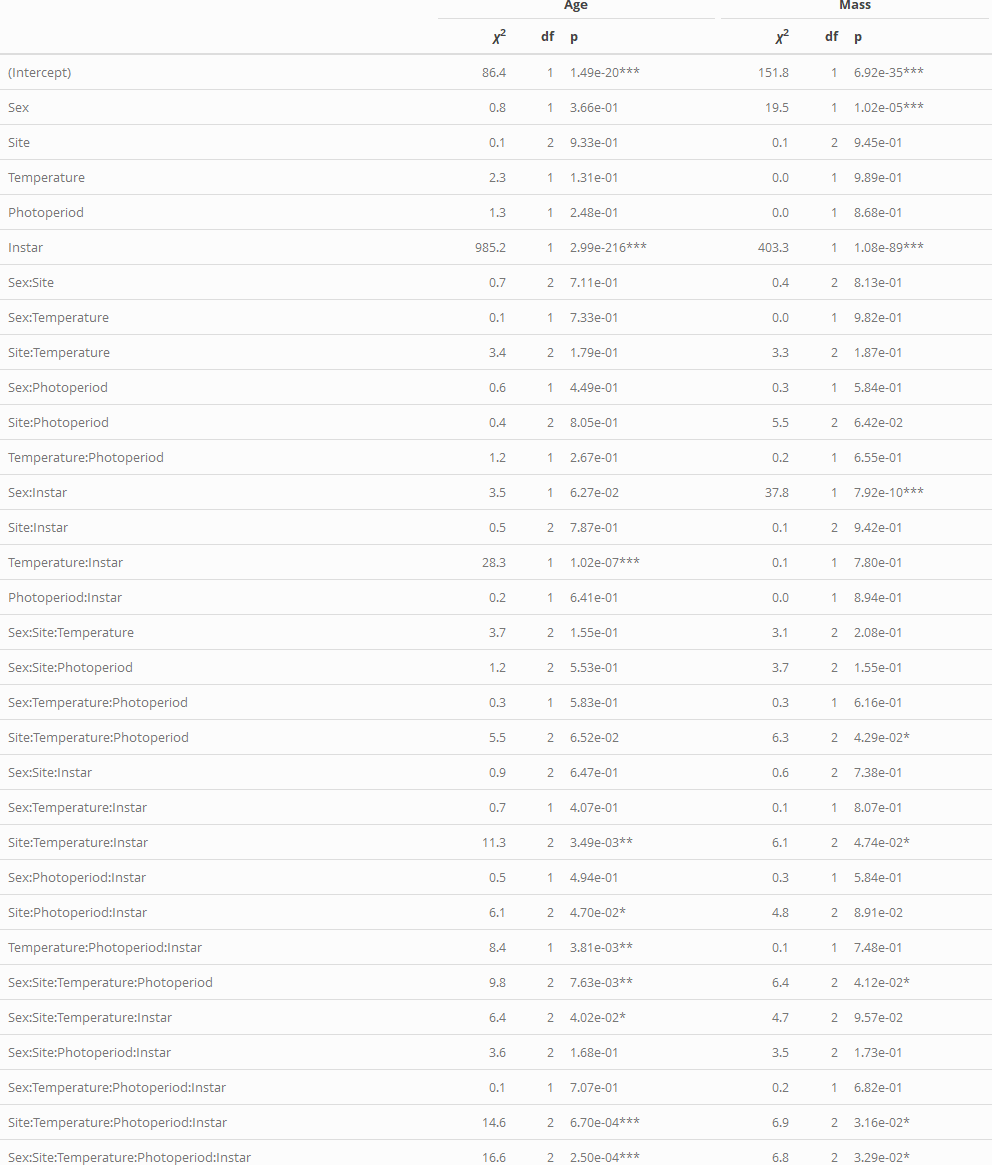


Table S3. Estimated coefficients from our linear mixed effects model of grasshopper time to arrival as a function of which instar the grasshopper is arriving at, sex, photoperiod, site, and temperature variance. The intercept occurs when Sex = female, Temperature = high variance, Photoperiod = long, Site = 2195m, and Instar=0 (in our data instar values range from 3 (3rd instar) to 6 (Adult)). Stars indicate significant effects (*: p < 0.05, **: p < 0.01, and ***: p < 0.001).

*
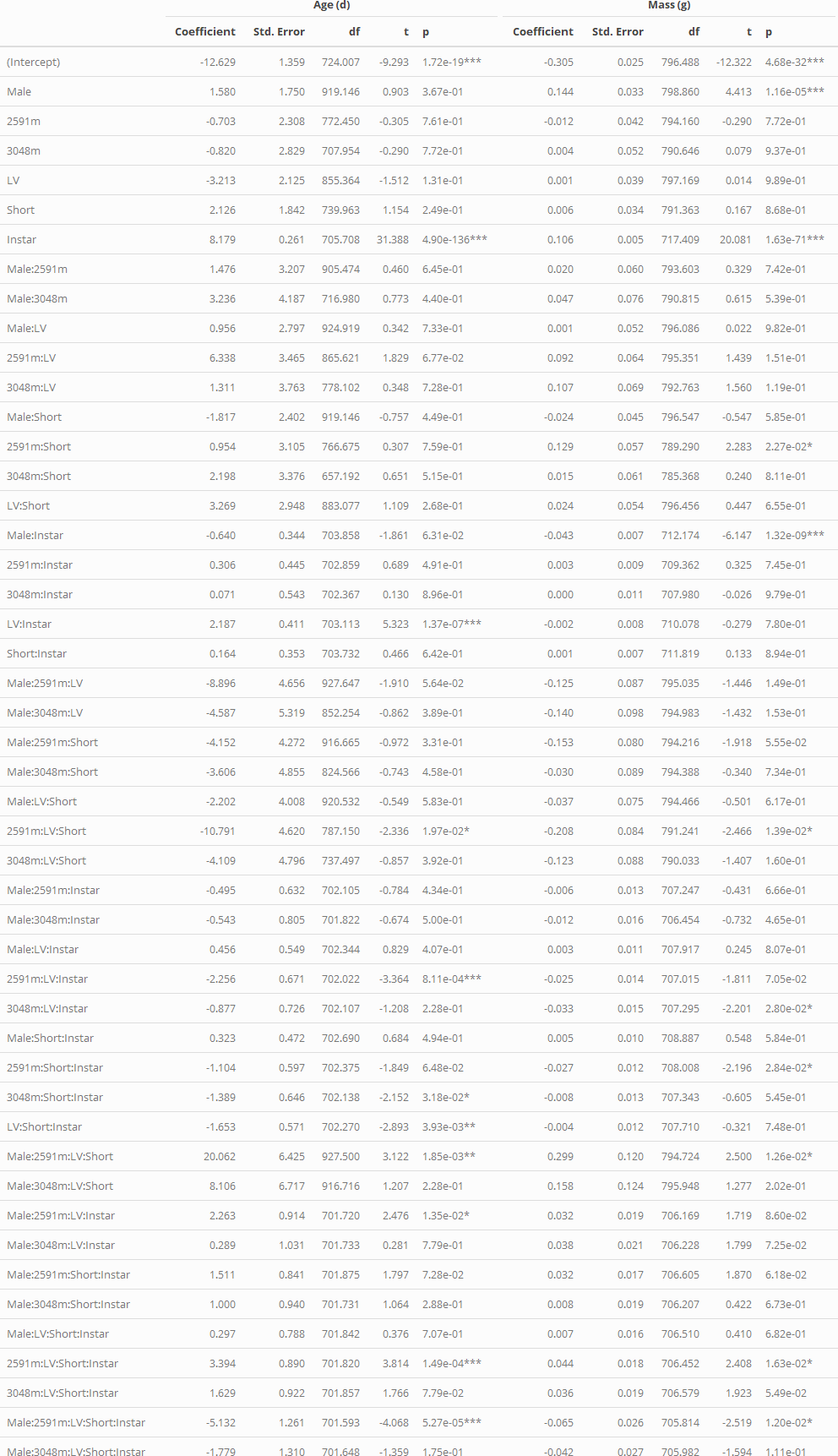
*

Table S4. Estimated coefficients from our linear mixed effects model of grasshopper preferred body temperature (in °C) as a function of sex, site, temperature variance, and photoperiod. Stars indicate significant effects (*: p < 0.05, **: p < 0.01, and ***: p < 0.001).

*
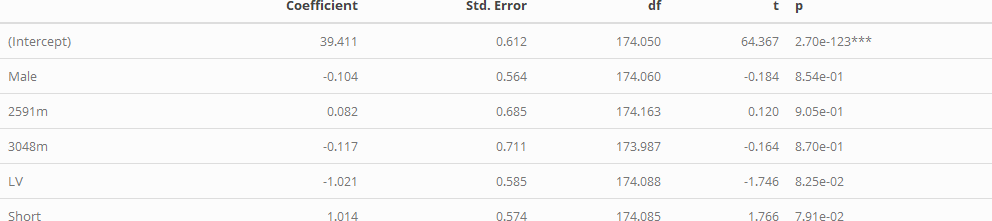
*

Table S5. Estimated coefficients from our linear models of grasshopper CTmin and CTmax as a function of temperature variance, photoperiod, and the interaction between the two. Stars indicate significant effects (*: p < 0.05, **: p < 0.01, and ***: p < 0.001).

*
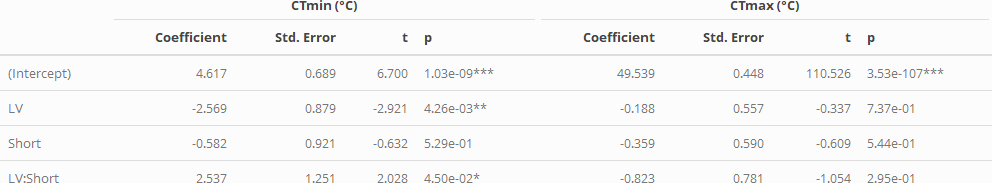
*

Table S6. Estimated coefficients from our linear mixed effects model of hopping and feeding performance. Stars indicate significant effects (*: p < 0.05, **: p < 0.01, and ***: p < 0.001).

*
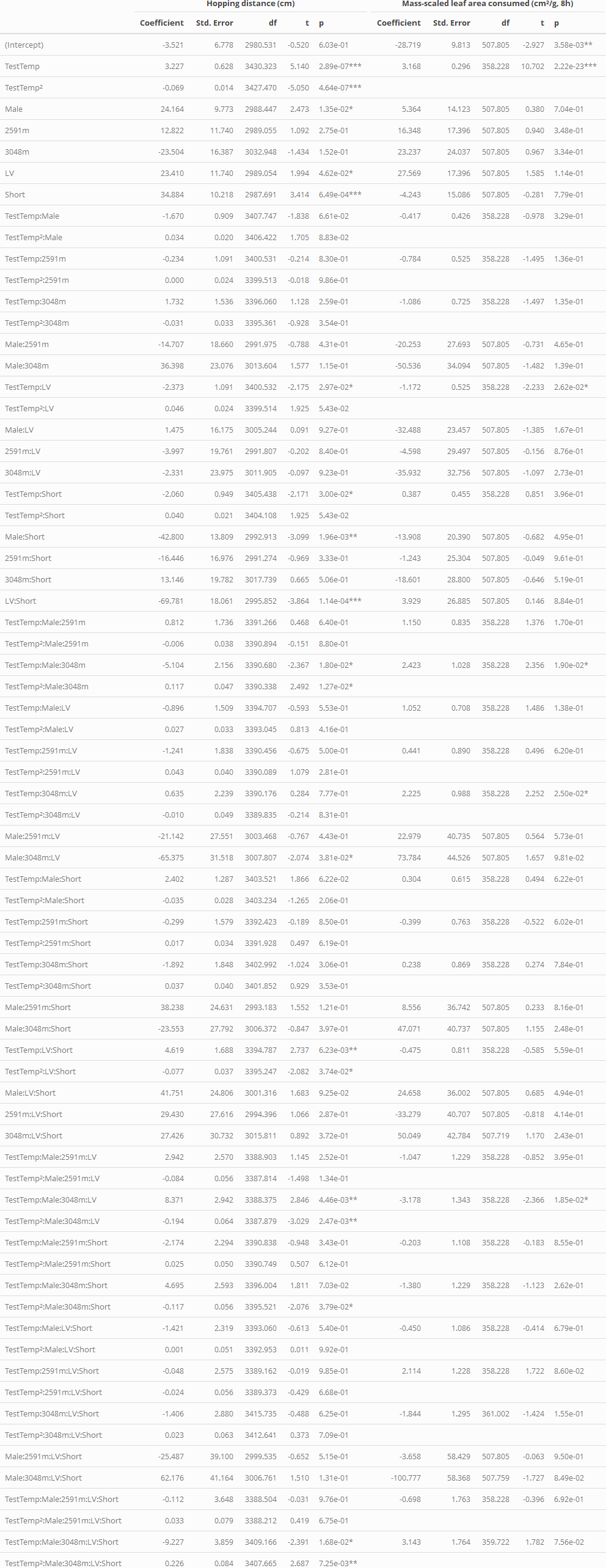
*

*
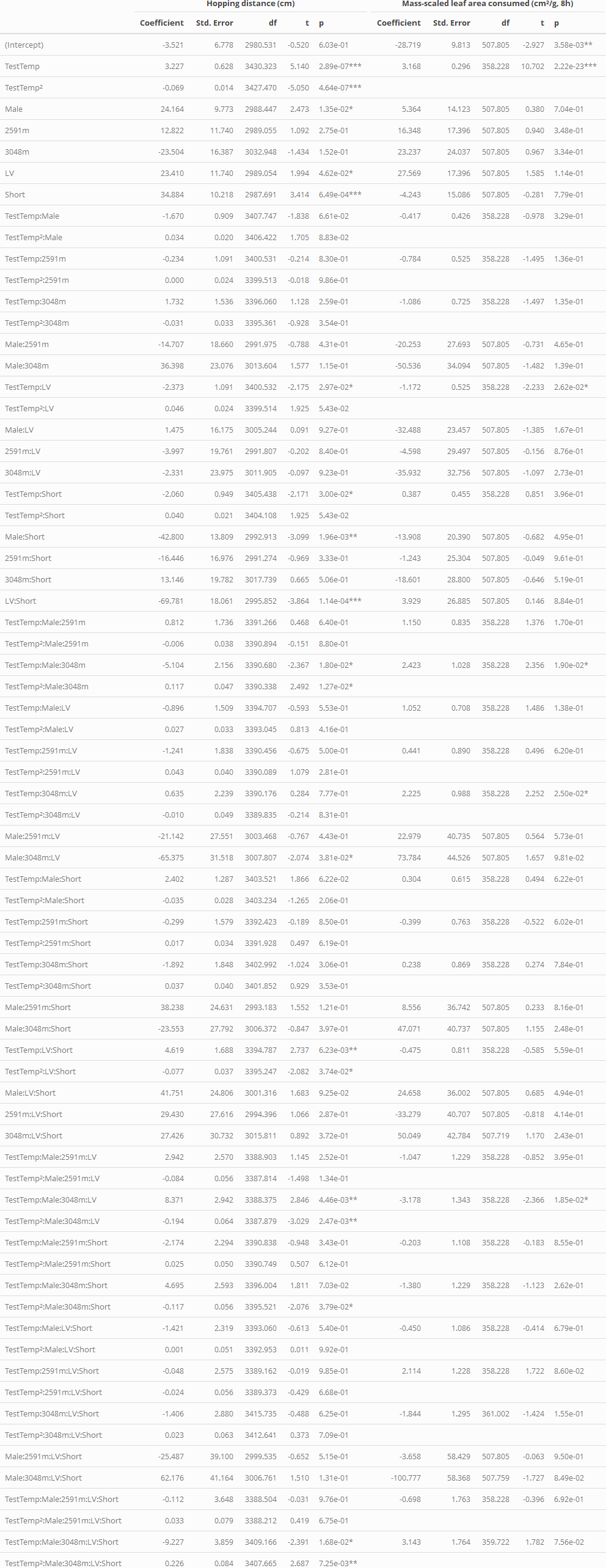
*


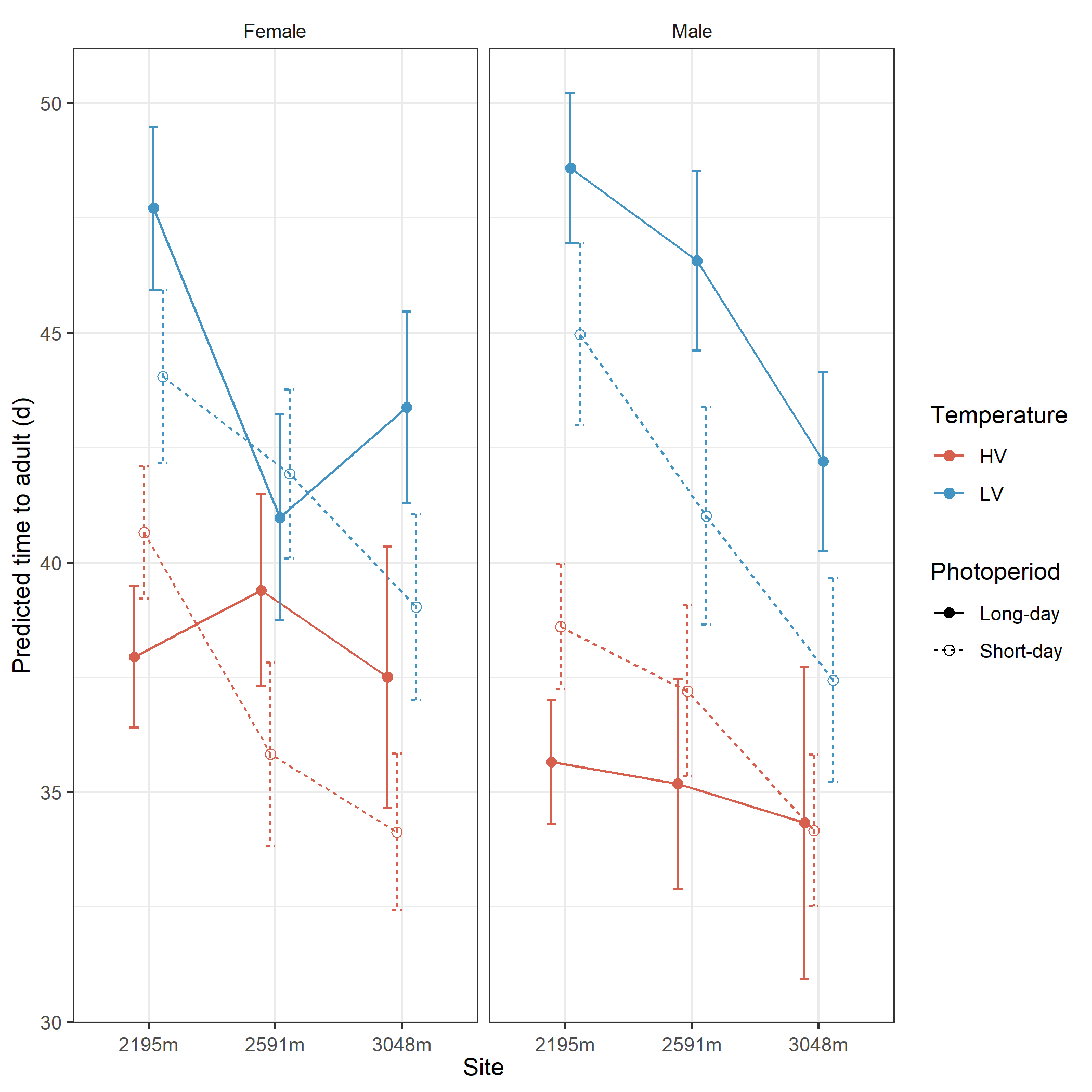


Fig S1. The significant fourth-order interaction effect from our linear mixed effects model of grasshopper time to adulthood (predictions are plotted). In long-day treatments, there is a greater discrepancy between high and low variance temperatures in high-elevation males than in high-elevation females.


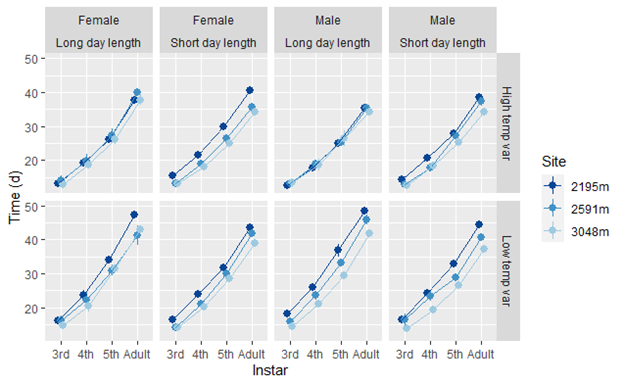


Fig S2. Time to arrival at each instar as a function of sex, photoperiod, site, and temperature variance.

*
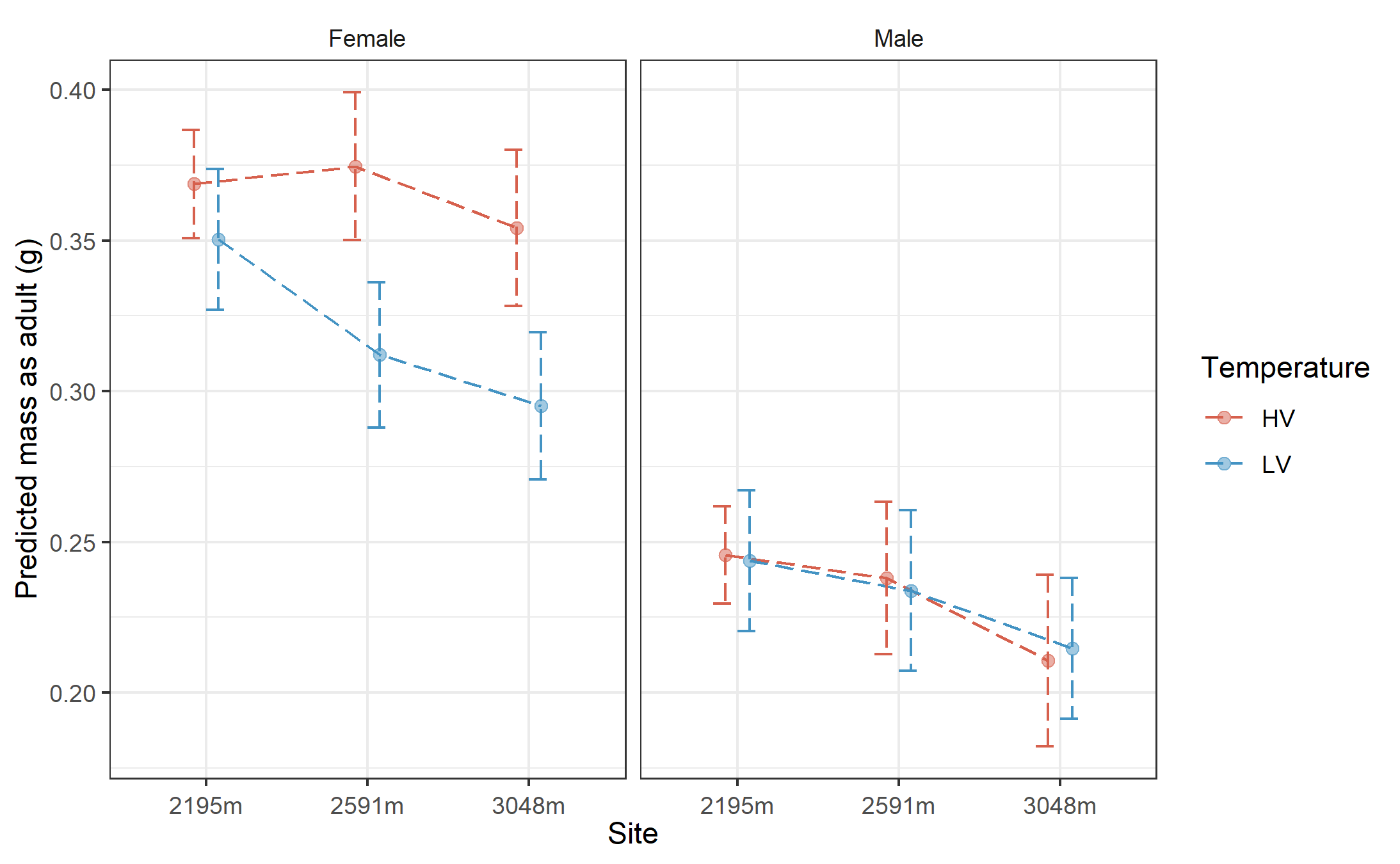
*

Fig S3. The significant sex-site-temperature interaction effect from our linear mixed effects model of grasshopper mass at adulthood (predictions are plotted). As site elevation goes up, the discrepancy between mass at high and low temperature variance grows as mass at low temperature variance declines – especially for females. In males, the high and low temperature variance masses look indistinguishable at each site.

*
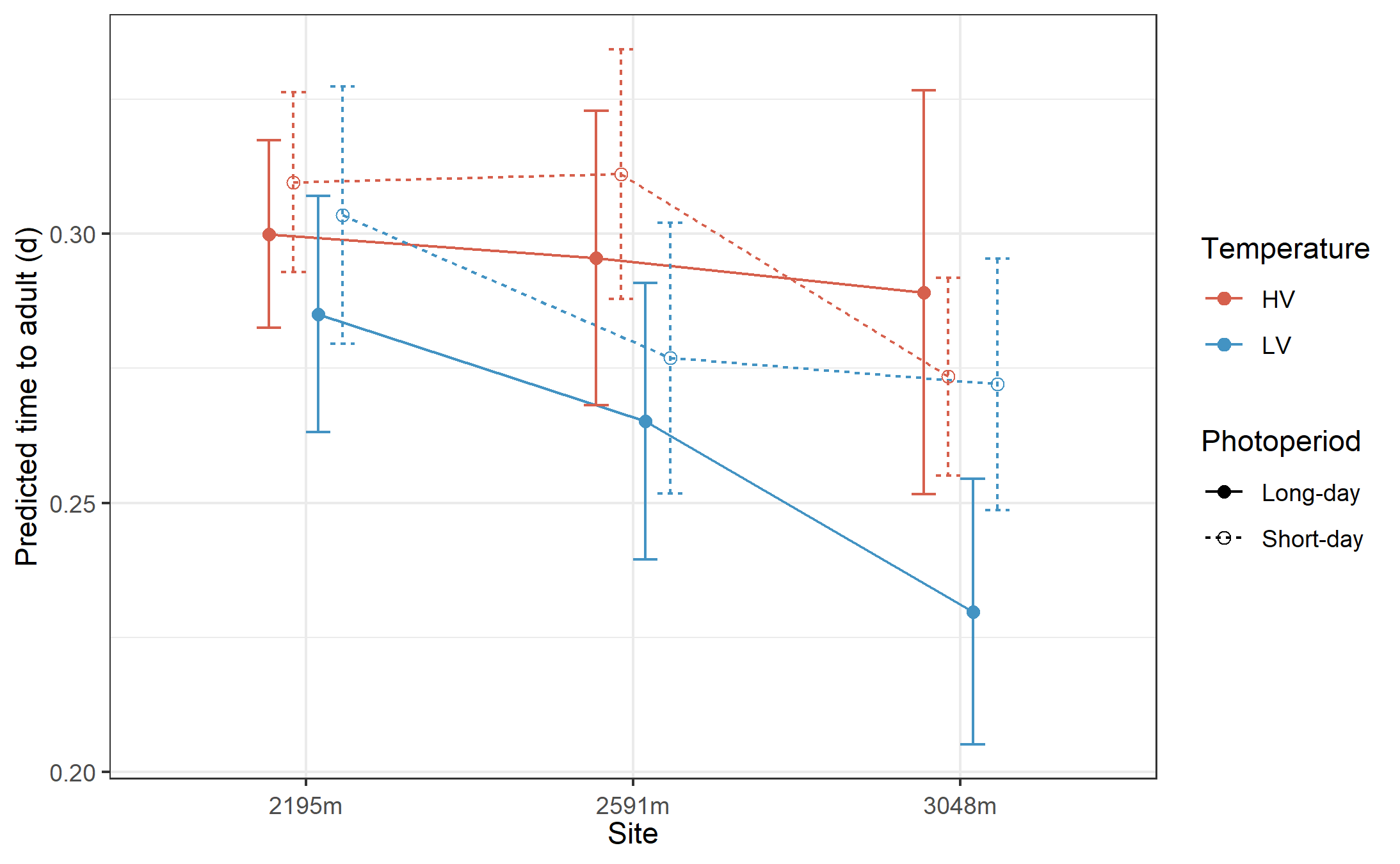
*

Fig S4. The nearly significant site-temperature-photoperiod interaction effect from our linear mixed effects model of grasshopper mass at adulthood (predictions are plotted). At long-day photoperiod, the discrepancy between high and low temperature variance grows as site elevation goes up as a result of declining masses at low variance. Note that the site-temperature-photoperiod interaction is not quite significant (χ^2^_2_=5.9, p=.0526, Table 1), but the trend is significant when considering only the 2195m and 3048m sites (t=2.381, p<.05, Table S1).


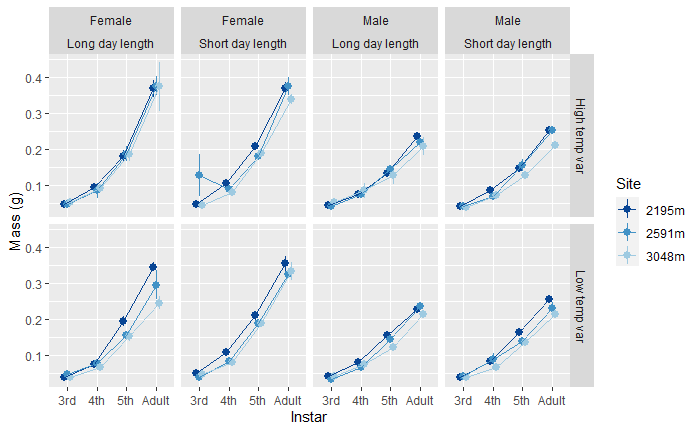


Fig S5. Mass at each instar as a function of sex, photoperiod, site, and temperature variance.
